# Three Novel Loci for Infant Head Circumference Identified by a Joint Association Analysis

**DOI:** 10.3389/fgene.2019.00947

**Published:** 2019-10-11

**Authors:** Xiao-Lin Yang, Shao-Yan Zhang, Hong Zhang, Xin-Tong Wei, Gui-Juan Feng, Yu-Fang Pei, Lei Zhang

**Affiliations:** ^1^Center for Genetic Epidemiology and Genomics, School of Public Health, Medical College, Soochow University, Jiangsu, China; ^2^Jiangsu Key Laboratory of Preventive and Translational Medicine for Geriatric Diseases, School of Public Health, Medical College, Soochow University, Jiangsu, China; ^3^Department of Epidemiology and Health Statistics, School of Public Health, Medical College, Soochow University, Jiangsu, China

**Keywords:** infant head circumference, genome-wide association study, 3q23, 7p15.3, 9q33.3, birth weight, birth length

## Abstract

As an important trait at birth, infant head circumference (HC) is associated with a variety of intelligence- and mental-related conditions. Despite being dominated by genetics, the mechanism underlying the variation of HC is poorly understood. Aiming to uncover the genetic basis of HC, we performed a genome-wide joint association analysis by integrating the genome-wide association summary statistics of HC with that of its two related traits, birth length and birth weight, using a recently developed integrative method, multitrait analysis of genome-wide association (MTAG), and performed *in silico* replication in an independent sample of intracranial volume (*N* = 26,577). We then conducted a series of bioinformatic investigations on the identified loci. Combining the evidence from both the MTAG analysis and the *in silico* replication, we identified three novel loci at the genome-wide significance level (α = 5.0 × 10^−8^): 3q23 [lead single nucleotide polymorphism (SNP) rs9846396, *p*_MTAG_ = 3.35 × 10^−8^, *p*_replication_ = 0.01], 7p15.3 (rs12534093, *p*_MTAG_ = 2.00 × 10^−8^, *p*_replication_ = 0.004), and 9q33.3 (rs7048271 *p*_MTAG_ = 9.23 × 10^−10^, *p*_replication_ = 1.14 × 10^−4^). Each of the three lead SNPs was associated with at least one of eight brain-related traits including intelligence and educational attainment. Credible risk variants, defined as those SNPs located within 500 kb of the lead SNP and with *p* values within two orders of magnitude of the lead SNP, were enriched in DNase I hypersensitive site region in brain. Nine candidate genes were prioritized at the three novel loci using multiple sources of information. Gene set enrichment analysis identified one associated pathway GO:0048009, which participates in the development of nervous system. Our findings provide useful insights into the genetic basis of HC and the relationship between brain growth and mental health.

## Introduction

The size of infant head circumference (HC) is thought to affect brain development in later life (Ivanovic et al., 2004). It is associated with a variety of intelligence- and mental-related conditions, such as intelligence quotient (IQ) score (Ivanovic et al., 2004), autism (Aylward et al., 2002), and Alzheimer’s disease (Schofield et al., 1997). Its relation to nonmental-related disorders, such as cardiovascular disease, is also reported (Barker et al., 1993). Therefore, understanding the mechanism of HC development is important in relation to its impact on phenotypic outcomes in later life in order to better prevent and intervene the related disorders.

Despite being influenced by maternal nutrition during gestation, infant HC is dominantly determined by inheritance (Smit et al., 2010). A previous twin study estimated its heritability to be as high as 0.7–0.9 (Smit et al., 2010). Early candidate gene studies have identified multiple rare genomic mutations with large effects (Bond et al., 2005; Kumar et al., 2009). For common variants, Taal et al. (2012) and Haworth et al. (2019)performed two genome-wide association study (GWAS) meta-analyses in European subjects and identified five loci (4q28.1, 6p21.21, 12q15, 12q24, and 17p13.1) in total. Nonetheless, variants identified by these studies explain only a small proportion of the total HC heritability, and the vast majority of missing heritability is yet to be discovered.

As a measure at birth, HC is related to birth length (BL) and birth weight (BW). They have robust links with each other in epidemiology and are all associated with common factors such as fetal sex, maternal factors, and gestational age (Yokoyama et al., 2005; Rydbeck et al., 2014). Many studies attempt to define some conceptual growth parameters by integrating the three traits to comprehensively reflect children’s health status (Sankilampi et al., 2013; Haksari et al., 2016). Their high phenotypic correlation (Scheffler et al., 2017) implies that these traits may share some common genetic architecture.

Recently, a new statistical method multitrait analysis of genome-wide association (MTAG) was proposed, which integrates the information contained in correlated traits to boost genetic association signals for each individual trait (Turley et al., 2018). In the present study, we will perform a joint genetic association analysis of HC, BL, and BW with MTAG. The main purpose is to identify novel genomic loci for HC by integrating the GWAS summary results from BL and BW. We will perform independent *in silico* replication in large-scale GWAS summary results of adult intracranial volume. We will then conduct genomic feature enrichment analysis and candidate gene prioritization. We will also perform gene set enrichment analysis to highlight relevant pathways.

## Materials and Methods

### Study Design

In this study, we performed a joint GWAS analysis by integrating the information carried in the GWAS summary results of HC, BL, and BW traits, with the main purpose of identifying responsible genomic loci for HC.

### Gwas Summary Results

All GWAS meta-analyses for the three individual traits were carried out by the Early Growth Genetics (EGG) consortium, and basic characteristics of individual studies are detailed in respective publications (**Table 1**) (Taal et al., 2012; van der Valk et al., 2015; Horikoshi et al., 2016). In brief, these three studies conducted meta-analyses in 28,459, 10,678, and 143,677 European participants for BL, HC, and BW, respectively. We accessed GWAS summary results from the consortium’s website (http://egg-consortium.org) and retrieved the part of results that included participants with European ancestry only for each trait.

**Table 1:** Basic characteristics of the three studied traits.

Trait	N	SNPs	Covariates	Ethnicity	H2 (s.e.)	Mean χ^2^	Intercept	Studies	Imputation reference panel	PMID
BL	28,459	2,201,903	Sex, age	European	0.169 (0.023)	1.067	0.992	22	HapMap Phase II	25281659
HC	10,768	2,449,806	Sex, age	European	0.227 (0.05)	1.041	0.991	7	HapMap Phase II	22504419
BW	143,677	16,245,523	Study-specific covariates	European	0.098 (0.007)	1.253	1.053	37	1000G project or combined 1000G and UK10K projects	27680694

N, sample size of each publication; Studies, the number of individual studies included into the meta-analysis; H2, mean chi-square and intercept were simultaneously estimated by LD Score regression at default settings, where s.e. means standard error of corresponding statistic.

### Single Nucleotide Polymorphism Inclusion Criteria

A series of sequential quality control (QC) criteria were applied to retain eligible single nucleotide polymorphisms (SNPs): 1) Duplicated SNPs were dropped within each trait (0 for BL, 2 for HC, 30,565 for BW); 2) SNP genomic coordinate for BL and HC was transformed from GRCH 36 to GRCh 37 with LiftOver (http://genome.ucsc.edu/cgi-bin/hgLiftOver), and deprecated SNPs were dropped (213 for BL and 256 for HC); 3) for BL summary results with no allele frequency information, allele frequency was estimated based on the HapMap Phase 2 + 3 European individual genotypes, and SNPs with missing allele frequency were excluded (Ivanovic et al., 2004, 748); 4) rare SNPs of minor allele frequency (MAF) below 1% were excluded within each trait (2,876 for BL and 6,665,395 for BW); 5) SNPs on which the sample size was smaller than 75% of 90th percentile of SNP-level sample size distribution were excluded within each trait (3,809 for BL, 36,610 for HC, and 10,607 for BW) (Turley et al., 2018); and 6) SNPs having nonconcordant alleles (e.g., A/G vs. A/C, 61) or with ambiguous strands (e.g., A/T and C/G, 168,789) across the three traits were excluded. After the above QC steps, there were 1,977,400 qualified SNPs in common to the three traits. All subsequent analyses were based on these SNPs.

### LDSC Analysis

We applied the linkage disequilibrium score (LDSC)-based regression analysis to the summary results of each trait to evaluate its genetic architecture. LDSC takes GWAS summary statistics as input and partitions overall inflated association statistics into one part attributable to polygenic architecture and another part due to cryptic relatedness and population stratification (Bulik-Sullivan et al., 2015b). LDSC can also estimate genetic heritability attributable to the GWAS SNPs. Reference LD scores for the European population were downloaded from the software website (https://github.com/bulik/ldsc). 

We also applied bivariate LDSC regression analysis implemented in LD-Hub (http://ldsc.broadinstitute.org/ldhub/) to the GWAS summary results of HC and BL traits, and of HC and BW traits, to study the genetic correlations between HC and the other two traits (Bulik-Sullivan et al., 2015a). In brief, this method is an extension of single-trait LD score regression and requires only GWAS summary statistics from individual studies, being robust to sample overlap.

### Joint Analysis by MTAG

We applied the MTAG method to the three traits for a joint association analysis (Turley et al., 2018). MTAG estimates per SNP effect size for each trait by incorporating information contained in the other correlated traits and therefore has potential to improve statistical power of association test. MTAG takes summary statistics from multiple studies as input. We used the 1,000 genomes project European individuals as reference panel for LD estimation for all the three traits. The effect size re-estimated by MTAG is a generalized estimate of inverse-variance-weighted meta-analysis by integrating association summary statistics from different traits, where *p* value is derived from the re-estimated effect size (Turley et al., 2018). A distinct locus was defined as a genomic region encompassing the lead SNP and its flanking 500-kb region in either direction.

### Variance Explained by SNPs

All the three traits are standardized (i.e., mean 0 and variance 1) in their original studies. Therefore, we estimated individual variant effect size as the variance it explains with the formula 2*f*(1 − *f*)β^2^, where *f *is allele frequency and β is regression coefficient.

### *In Silico* Replication in Intracranial Volume Summary Results

Each lead SNP from the newly identified loci was subject to independent *in silico* replication in intracranial volume. This is because HC is both phenotypically and genetically highly correlated with intracranial volume (Adams et al., 2016; Breakey et al., 2018). Their genetic correlation coefficient is reported to be as high as 0.75, indicating a shared genetic background (Adams et al., 2016). Some previous studies integrate these two traits together for a direct meta-analysis (Adams et al., 2016; Haworth et al., 2019).

The summary results of intracranial volume come from a large-scale GWAS meta-analysis conducted by the CHARGE and the ENIGMA consortia in 26,577 subjects of European ancestry and were downloaded through the ENIGMA’s website (http://enigma.ini.usc.edu/research/download-enigma-gwas-results/) with permission.

The effect direction was first compared between the discovery and the replication stages. Given consistent effect direction, one-sided *p* value from replication stage was then reported. To account for multiple testing problem, the Bonferroni correction was adopted to declare statistical significance in the replication stage.

### *In Silico* Replication of BL and BW

Although the focus of the present study was HC, it did produce re-estimated MTAG results for BL and BW. We performed *in silico* replication of these two traits to strengthen the validity of our analytical approach. For BL, we replicated in the large-scale GWAS summary results released by the GIANT consortium for adult height (Yengo et al., 2018) (http://portals.broadinstitute.org/collaboration/giant/index.php/GIANT_consortium_data_files), where adult height is believed to closely link to the BL (van der Valk et al., 2015). For BW, we replicated in BW GWAS summary results conducted in ∼330,000 UK Biobank participants. The analysis was performed by Dr. Neale’s lab, and the results were kindly released to be publicly available at the lab website (http://www.nealelab.is/uk-biobank). We kept in mind that about 67,000 participants overlapped between this analysis and the BW results used in the present study. Again, the replication significance was evaluated in terms of both effect direction and Bonferroni corrected one-sided *p* value.

### Association With Brain/Mental-Related Traits

We explored the association of the SNPs identified in the present study for HC with multiple mental health and intelligence-related traits. A total of eight traits were collected, which could be classified into two categories. The first category contains two normal traits intelligence (Savage et al., 2018) and educational attainment (Lee et al., 2018). The second category contains the remaining six disease-related traits, including subjective wellbeing (SWB) (Okbay et al., 2016), broad depression (Howard et al., 2018), autism spectrum disorder (ASD) (Grove et al., 2017), loneliness (http://www.nealelab.is/uk-biobank), cognitive impairment (CI) (http://www.nealelab.is/uk-biobank) and Parkinson disease (PD) (http://www.nealelab.is/uk-biobank). 

### Functional Enrichment Analysis

We assessed the enrichment of the HC SNPs into different genomic features with the GWAS Analysis of Regulatory or Functional Information Enrichment with LD correction (GARFIELD) (Iotchkova et al., 2016). GARFIELD employs an adaptive permutation procedure that creates and utilizes a set of null variant that accounts for systematic differences in minor allele frequency, gene distance, and number of proxies in the test variant set (Iotchkova et al., 2016).

Genomic feature information was accessed from the software website (https://www.ebi.ac.uk/birney-srv/GARFIELD/). Genomic features include DNase I hypersensitive sites and genic features from three tissues (fetal brain/brain, fetal hippocampus, and cerebellum) to capture the cell-type-specific feature enrichment. The assessment was conducted at four *p* value thresholds (1.0 × 10^−5^, 1.0 × 10^−6^, 1.0 × 10^−7^, and 5.0 × 10^−8^). To account for correlation between genomic features, the effective number of genomic features was first estimated by GARFIELD, which was 35.32. Statistical significance was then declared at a Bonferroni-corrected threshold, i.e., 1.42 × 10^−3^ (0.05/35.32).

### Candidate Gene Prioritization

Within each locus, we first defined a set of credible risk variants (CRVs) as those located within 500 kb of the lead SNP, and with *p* values within two orders of magnitude of the lead SNP (Michailidou et al., 2017). This is approximately equivalent to defining CRVs whose posterior probability of causality is within two orders of magnitude of the value of the lead SNP (Udler et al., 2010).

We incorporated five sources of information to seek evidence of a gene’s causality: i) being the gene nearest the lead CRV, ii) containing a missense coding CRV, (iii) being the target gene for any cis-eQTL CRV, iv) being prioritized by DEPICT, or v) being prioritized by SMR analysis.

Cis-eQTL information was obtained from two datasets: GTEx (v7) (https://gtexportal.org/home/) (GTEx, 2017) and the study of Westra et al. (Westra et al., 2013). Cis-eQTL information for GTEx is available for over 50 tissues, and for Westra et al., it is only available for whole blood. We explored all tissues to avoid missing a tissue-specific eQTL. We used the web tool HaploReg v4.1 (Ward et al., 2012) to annotate missense coding variants and cis-eQTLs (https://pubs.broadinstitute.org/mammals/haploreg/haploreg.php). Significance threshold was set at 0.05.

DEPICT is an integrative tool that employs predicted gene functions to systematically prioritize the most likely causal genes at associated loci (Pers et al., 2015). Significant genes were declared at a false discovery rate (FDR) = 0.05.

SMR is another prioritization method that integrates summary-level data from GWAS with cis-eQTL data to identify genes whose expression levels are associated with trait because of pleiotropy (Zhu et al., 2016). Three cis-eQTL datasets were analyzed. The first was the study of Westra et al. (Westra et al., 2013), and the second and third ones were two brain-related tissues from Qi et al. brain eQTL results and the PsychENCODE dataset (Gandal et al., 2018; Qi et al., 2018). All cis-eQTL datasets were downloaded from the SMR website. The LD reference panel required by SMR was formed by the 503 European subject haplotypes of the 1000G project. Specifically, the length of the reference panel was 2 mega-base (MB) and was centered at the target lead SNP. Significance threshold was set at 0.05.

### Gene Set Enrichment Analysis

We performed gene set enrichment analysis with MAGMA (v1.6) (de Leeuw et al., 2015). MAGMA simultaneously analyzes multiple genetic markers incorporating LD between markers to determine their joint effect utilizing either summary data or individual-level data (de Leeuw et al., 2015). The total SNPs were mapped to 16,645 genes according to de Leeuw et al. (2015)and these genes were mapped to 9,467 predefined gene sets retrieved from GO consortium (http://www.geneontology.org/). A gene-based association test was examined to determine individual gene association signals. A gene set association signal was then calculated by integrating all gene signals within the set. We used competitive model to test whether genes in a gene set are more strongly associated with the phenotype than other genes and multitesting problem was corrected by permutation test.

## Results

### Joint Association Analysis

After a series of inclusion criteria, we finally included 1,977,400 SNPs for analysis. Using HC summary association statistics at these SNPs, the LDSC estimated a SNP attributable heritability 0.23 (s.e. = 0.05) for the trait. Meanwhile, the SNP attributable heritability for BL and BW was 0.169 and 0.098, respectively. There are two loci 12q15 and 12q24 being significant at the genome-wide significance level in the original HC summary results. After excluding the two lead SNPs (rs1042725 and rs7980687) and their neighboring SNPs within 500 kb to either direction, the heritability is 0.22 (s.e. = 0.04), showing that most of the SNP-attributable heritability is hidden in the current GWAS setting.

When applied a LDSC-based genetic correlation analysis, the genetic correlation coefficient between HC and BL is estimated to be as high as 0.55 (s.e. = 0.12) and that between HC and BW is 0.37 (s.e. = 0.08), in line with previous research (Horikoshi et al., 2016) (**Table 2**). This implies that the three traits have shared genetic background.

**Table 2:** Genetic correlations of head circumference (HC) and other two related traits.

Trait	BL(s.e.)	HC(s.e.)	BW(s.e.)
BL(s.e.)	–	0.55 (0.12)	0.81 (0.06)
HC(s.e.)	0.55 (0.12)	–	0.37 (0.08)
BW(s.e.)	0.81 (0.06)	0.37 (0.08)	–

Genetic correlation, representing the proportion of the total genetic variance that two traits share, was estimated by LDSC software using summary results. s.e., standard error of genetic correlation.

We then applied the MTAG method to the three studies for a joint analysis. Briefly, MTAG jointly analyzes GWAS summary statistics from different traits, possibly from overlapping samples, and reports association results for each trait. In the MTAG analysis of the HC trait, the LDSC intercept and mean chi-square are 0.99 and 1.04, suggesting that the vast majority of inflation in the mean chi-squared statistic is from polygenic architecture rather than from population stratification.

The two originally significant loci remain significant at the genome-wide significance level (GWS, 5 × 10^−8^), with the exact same two lead SNPs (rs1042725 and rs7980687) and stronger signals. To search for additional loci, we evaluated MTAG results of all SNPs excluding those within 500 kb of a locus that was reported previously. These identified 76 SNPs in 9 distinct loci that are significant at the GWS level. Of the nine lead SNPs, four are not significant in the original HC study at the nominal level (α = 0.05) but extremely significant for BW and BL, indicating that the significant signals are likely to be driven solely by BL and/or BW instead by HC. As a QC criterion, we discarded these four loci.

**Figure 1:**
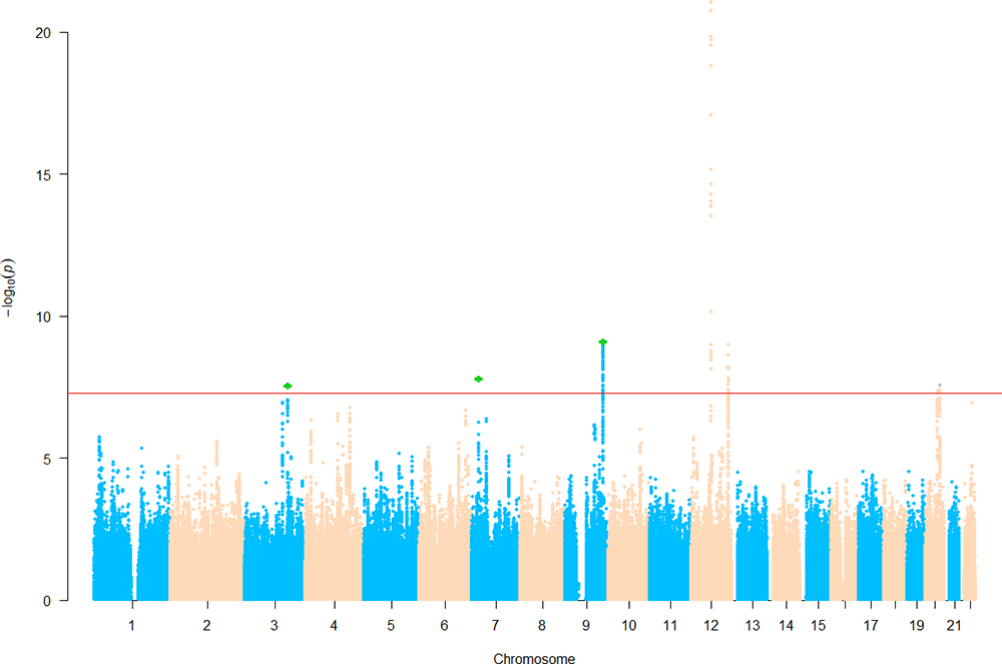
Manhattan plot of head circumference (HC). Known loci were retrieved from the EBI GWAS catalog website. Novel single nucleotide polymorphisms (SNPs) below the significance threshold 5.0 × 10^−8^ are marked in green.

We then sought for *in silico* replication of the identified SNPs in an independent large-scale GWAS meta-analysis of intracranial volume (*N* = 26,577). Both previously identified SNPs are significant (one-sided *p* = 3.22 × 10^−9^ and 0.03) in the replication results, demonstrating their robustness. Of the five newly identified loci, all are consistent in effect direction between HC and intracranial volume. Among them, one-sided *p* value at three SNPs further achieves the Bonferroni-corrected significance level (α = 0.01), while rs3795128 has a weaker but nonsignificant signal (*p* = 0.02). Taken together, SNPs from three loci were convincingly associated with HC in both discovery and replication stages. The three lead SNPs explain 0.6% of HC phenotypic variance. Results are listed in **Table 3** and **Supplementary Table 1**, and the Manhattan plot is displayed in **Figure 1**.

**Table 3:** The main results of the identified loci for HC.

rs#	Chr	Pos	Locus	Alleles	FRQ	Original			MTAG			Replication	
						Beta	SE	P	Beta	SE	P	Z	*P* (one-side)
Known													
rs1042725	12	66358347	12q14.3	T/C	0.49	-0.10	0.01	6.58 × 10^−7^	−0.1	0.01	8.55 × 10^−22^	−5.81	3.22 × 10^−9^
rs7980687	12	123822711	12q24.31	A/G	0.20	0.09	0.02	3.34 × 10^−7^	0.08	0.01	1.02 × 10^−9^	1.91	0.025
Novel													
rs9846396	3	141140968	3q23	T/C	0.46	0.05	0.01	2.60 × 10^−4^	0.06	0.01	3.35 × 10^−8^	2.26	0.01
rs12534093	7	23502974	7p15.3	A/T	0.23	−0.06	0.02	6.23 × 10^−4^	−0.08	0.01	2.00 × 10^−8^	−2.65	0.004
rs7048271	9	125918772	9q33.3	A/G	0.15	-0.10	0.02	6.71 × 10^−4^	0.1	0.02	9.23 × 10^−10^	3.69	1.14 × 10^−4^

Chr, chromosome; Pos, genomic position based on the GRCH37 genome assembly; the first/second alleles are the effect and alternative alleles. FRQ, allele frequency of effect allele; Beta, regression coefficient; SE, standard error of beta; Z, z-score of beta; P, p value.

For BL and BW, new loci beyond the originally significant ones are identified, too. For BL, a total of 539 SNPs from 27 distinct loci are significant at the GWS level. Of them, only one was reported by the original study, while the remaining 26 loci are novel (**Supplementary Table 2**). For BW, a total of 731 SNPs from 40 distinct loci are significant at the GWS level. Of them, 39 loci are significant in the original BW results, and the remaining one locus 10q22.1 is novel (**Supplementary Table 2**). We also performed *in silico* replications in adult height and BW, respectively. As shown in **Supplementary Table 2**, up to 21 of the 26 newly identified loci for BL are significant in adult height, and the only one newly identified locus for BW is significant in the UK Biobank BW. These largely replicated results further strength the validity of the present integrative analysis.

### Association With Brain/Mental Related Traits

We next evaluated the relation of the five identified HC SNPs (two known + three new) to several mental health and intelligence-related traits and diseases. We collected GWAS summary results for eight traits. *p* values of the five lead SNPs in these traits are listed in **Table 4**.

**Table 4:** The association of head circumference (HC) single nucleotide polymorphisms (SNPs) with other mental traits.

SNP	Locus	Intelligence	EA	SWB	Loneliness	Depression	ASD	CI	PD
Known									
rs1042725	12q14.3	1.1 × 10^−3^	**7.63 × 10** **^−6^**	**6.63 × 10** **^−4^**	0.03	0.02	0.23	0.88	0.03
rs7980687	12q24.31	**1.44 × 10** **^−6^**	**3.01 × 10** **^−25^**	0.51	0.11	0.01	0.90	0.44	0.54
Novel									
rs9846396	3q23	0.46	**1.44 × 10** **^−7^**	0.92	0.40	0.78	0.73	0.27	0.70
rs12534093	7p15.3	**3.25 × 10** **^−4^**	0.13	0.78	9.74 × 10^−3^	0.54	0.22	0.42	0.36
rs7048271	9q33.3	0.53	0.22	0.02	**1.82 × 10** **^−4^**	0.10	0.05	0.56	0.74

EA, educational attainment; SWB, subjective well-being; ASD, autism spectrum disorder; CI, cognitive impairment, including dementia and Alzheimer’s disease; PD, Parkinson’s disease. p values significant after multiple testing correction (*α = *1.25 × 10^−3^) were marked in bold.

At the Bonferroni corrected significance level (0.05/40 = 1.25 × 10^−3^), any of the five SNPs is associated with at least one trait. The two known SNPs rs1042725 and rs7980687 are both associated with educational attainment (rs1042725 *p* = 7.63 × 10^−6^ and rs7980687 *p* = 3.01 × 10^−25^). In addition, rs1042725 is associated with subjective well-being (*p* = 6.63 × 10^−4^), and rs7980687 is associated with intelligence (*p* = 1.44 × 10^−6^). The three newly identified SNPs are associated with educational attainment (rs9846396 *p* = 1.44 × 10^−7^), intelligence (rs12534093 *p* = 3.25 × 10^−4^) or loneliness (rs7048271 *p* = 1.82 × 10^−4^). There is also a hint for the association of rs12534093 with loneliness, although the signal (*p* = 9.74 × 10^−3^) does not reach the significance threshold.

Notably, the three loci (3q23, 9q33.3, and 12q24.31) associated with educational attainment have been identified to be significant at the GWS level (Savage et al., 2018), with different lead SNPs. At 3q23 and 12q24.31, the lead SNPs in the present study are in high linkage disequilibrium (0.81–0.98) with the GWS lead SNPs, respectively, indicating that both signals arise from the same association signal at respective locus.

### SNP Enrichment in Genomic Features

We investigated the enrichment of the identified SNPs into a variety of genomic features in three brain-related tissues including brain/fetal brain, fetal hippocampus, and cerebellum. The results are shown in **Supplementary Table 3**. At the most stringent threshold (*T* = 5.0 × 10^−8^), significant enrichment is observed in up to 13 features. Notably, SNPs are enriched in the DNase I hypersensitive site peak and a broader DNase I hypersensitive site category hotspot in only one (brain/fetal brain, OR: 10.71–30.44, *p*: 1.66 × 10^−6^–2.79 × 10^−4^) of the three tissues being investigated, but not in fetal hippocampus and cerebellum. At footprint feature, which is defined as a paucity of cleavage because protein-bound DNA is typically protected from DNase I cleavage, SNPs are observed to be enriched in brain (OR = 12.11, *p* = 7.62 × 10^−4^) and brain hippocampus (OR = 12.26, *p* = 3.39 × 10^−4^). SNPs are also enriched in exon (OR = 10.94, *p* = 1.21 × 10^−3^) and intergenic regions (OR = 10.70, *p* = 1.33 × 10^−3^) with lower ORs. At *T* = 1.0 × 10^−7^, significant enrichment is found in the same 13 features with similar ORs. At T = 1.0 × 10^−6^ and 1.0 × 10^−5^, however, enrichment is observed in a fewer number of features (9 and 0, respectively).

### Candidate Gene Prioritization

We combined five sources of information to prioritize responsible candidate genes. The results are listed in **Table 5**. Sample size for the several tissues being analyzed varies from 175 to 491, all greater than the median across the total 53 tissues. A total of nine genes are prioritized. Among them, two genes (*ZBTB38* and *STRBP*) are prioritized by more than one source. Both genes are closest to respectively lead SNPs and are the cis-eQTL target gene of the latter. What is more, previous research demonstrates that *STRBP* at 9q33.3 is differentially expressed between patients with Down’s syndrome and controls (Salemi et al., 2012).

**Table 5:** Prioritized candidate genes at the identified novel loci.

SNP	Locus	Gene
rs9846396	3q23	*ZBTB38* (C, N, S), *XRN1*(S)
rs12534093	7p15.3	*IGF2BP3* (N), *STEAP1B* (S), *DFNA5 *(S)
rs7048271	9q33.3	*STRBP* (C, N), *PTGS1* (S), *RABGAP1* (C), *ZBTB6* (S)

Genes are prioritized as following: gene nearest to the lead credible risk variant (CRV) (N); gene containing a missense CRV (M); gene with messenger RNA (mRNA) levels in association with one or more CRVs (cis-eQTL, C); gene prioritized by DEPICT(D); or gene prioritized by SMR analysis (S).

### Gene Set Enrichment Analysis

Based on genome-wide SNP associations, we performed gene set enrichment analysis. Two GO terms, GO:0048009 and GO:0003680, are significant after permutation-based multiple-testing correction (**Table 6**). The term GO:0003680 consists of two genes *KMT2A* and *HMGA2* whose *p* values are 0.15 and 2.55 × 10^−11^ in gene-based association results. Therefore, the signal of this gene set is presumably dominated by *HMGA2*. GO:0048009 is the series of molecular signals generated as a consequence of the insulin-like growth factor (IGF) receptor binding to one of its physiological ligands (http://amigo.geneontology.org/amigo/). The role of this gene set includes regulating the development and growth of central nervous system (O’Kusky et al., 2012). It may also contribute to the development of cognitive disorders (Xie et al., 2014; Pomytkin et al., 2018).

**Table 6:** Main results of gene set enrichment for head circumference (HC).

Term	Annotation	Genes	P	P_adj_
GO:0048009	Insulin-like growth factor receptor signaling pathway	*EIF2AK3, IRS1, GHR, PIK3R1, GRB10, GIGYF1, AKT1, IGF1R, TSC2*	1.20 × 10^−7^	0.04
GO:0003680	Interacting selectively and non-covalently with oligo and oligo tracts of DNA	*KMT2A, HMGA2*	3.71 × 10^−8^	0.03

GO terms were retrieved from the Gene Ontology consortium. P_adj_, p value after multiple testing adjustment by permutation.

## Discussion

In this study, we have performed a joint association analysis of HC and its two related traits BL and BW. We have identified three novel loci that are successfully replicated by intracranial volume in an independent large-scale study, increasing the number of associated loci from five to eight. We have also evaluated the enrichment of the associated SNPs into multiple genomic features, prioritized candidate genes, and identified responsible gene sets.

Our MTAG analysis identified four HC loci at the GWS level that are not significant in the original HC study even at the nominal level (α = 0.05). As a QC criterion, we discarded these four loci because they might represent false positive signals. As stated by the authors of MTAG, this false positive finding could happen when SNP is nonsignificant for one trait but significant for others in finite samples. Indeed, none of these SNPs could be replicated by intracranial volume (**Supplementary Table 2**), strengthening the possibility of false positive signals.

We could not replicate rs3795128 (20q12) in intracranial volume at Bonferroni-corrected threshold, although it was nominally significant (one-sided *p = *0.02) in this dataset. We prioritized four genes (*PLCG1*, *LPIN3*, *ZHX3*, and *TOP1*) for this SNP using same gene prioritization strategy. Among them, *PLCG1* is the most promising candidate gene at 20q12 detected by multiple sources because it is the closest to lead CRV (rs3795128) and contains a missense CRV (rs753381) that is in high linkage equilibrium (*r* ^2^ = 0.7, 23 kb) with lead CRV. In a previous case–control study, missense mutations in *PLCG1* were reported to participate in the pathogenesis of cutaneous T-cell lymphomas by enhancing downstream signaling towards nuclear factor of activated T-cell activation. We therefore hypothesized that rs3795128 was linked to T-cell lymphomas through its strong LD with the missense mutation in *PLCG1* (Vaque et al., 2014). Evident impact of *PLCG1* is repeatedly reported in learning and memory through various pathways in hippocampal tissue in mouse model (Gruart et al., 2007; Yang et al., 2017). These clues linking HC with neurological diseases imply that early brain growth may have a connection with cognitive ability. Therefore, *PLCG1* may have a comprehensive regulatory effect on fetal growth and affect brain development in later life. Another candidate gene *STRBP* at 9q33.3 is reported to have a slight association with late-onset AD (Herold et al., 2016).

Given the connection between HC and cognitive ability and intelligence in epidemiology (Mosing et al., 2018), we explored this relation by investigating the association of the SNPs identified in the present study with brain health and intelligence-related traits/diseases. We observed an excess of significant SNPs in several traits including intelligence and educational attainment, in consistent with previous studies (Ivanovic et al., 2004).

The associated gene set GO:0048009 is an IGF receptor signaling pathway. In conjunction with its glycoprotein receptor (IGF-1R), IGF-1 is a phylogenetically ancient neurotrophic hormone and plays crucial roles in central nervous system development and maturation (Dyer et al., 2016). In astrocyte from mice, two previous studies demonstrated that cognition is influenced by IGF-1 by exhibiting expression of IGF-1R (Logan et al., 2018; Chen et al., 2019), as supported by both in animal and in clinical experiments (Baker et al., 2012; Andersson et al., 2018).

Certain limitations exist in the present study. First, the identified novel SNPs lack independent replication in the same trait. This is partly due to the relatively limited GWAS for HC in the community. As a compromise, we performed *in silico* replication in a genetically highly correlated trait intracranial volume. We believe that the replication results imply important clues towards true associations. Second, the unbalanced sample sizes used in the MTAG analysis may elevate false positive rate, as described by the MTAG authors (Scheffler et al., 2017). We tackled this issue by monitoring both the original and the integrative association signals for any unusual difference (e.g., non- to extreme significance). We also conducted an in-depth evaluation towards the relevance of the identified loci from a variety of sources, such as bioinformatical and functional investigation.

In summary, by integrating the GWAS summary statistics of HC, BL, and BW, we have performed a joint association analysis and identified three novel loci for HC. Not only that, 21 novel loci for BL and 1 for BW were also identified and replicated. We have also prioritized candidate genes and performed gene set enrichment analyses. Our results provide useful insights into the genetic architecture underlying HC and the development of its related mental conditions.

## Data Availability Statement

Publicly available datasets were analyzed in this study. These data can be found here: http://www.gefos.org and http://egg-consortium.org.

## Author Contributions

In this paper, X-LY is in charge of conceptualization/design, methodology, investigation, formal analysis, and resources. S-YZ contributed to methodology, formal analysis and funding for this study. HZ, X-TW and G-JF, are also co-authors in charge of conceptualization/design and data curation. The two corresponding authors, Y-FP and LZ are responsible for methodology, formal analysis, supervision/oversight, funding, and a final approval of the vision to be published.

## Funding

This study was partially supported by the funding from National Natural Science Foundation of China (31571291 to LZ, 31771417 and 31501026 to Y-FP), the Natural Science Foundation of Jiangsu Province of China (BK20150323 to Y-FP), a project funded by the Priority Academic Program Development of Jiangsu higher education institutions (PAPD), and the Undergraduate Innovation Program of Jiangsu Province (KY2019881B to X-LY).

## Conflict of Interest

The authors declare that the research was conducted in the absence of any commercial or financial relationships that could be construed as a potential conflict of interest.
